# Cerebellar control of thalamocortical circuits for cognitive function: A review of pathways and a proposed mechanism

**DOI:** 10.3389/fnsys.2023.1126508

**Published:** 2023-03-30

**Authors:** Detlef H. Heck, Mia B. Fox, Brittany Correia Chapman, Samuel S. McAfee, Yu Liu

**Affiliations:** ^1^Department of Biomedical Sciences, University of Minnesota Medical School, Duluth, MN, United States; ^2^Department of Anatomy and Neurobiology, College of Medicine, University of Tennessee Health Science Center, Memphis, TN, United States; ^3^Department of Neuroscience, University of Pennsylvania Perelman School of Medicine, Philadelphia, PA, United States; ^4^St. Jude Children’s Research Hospital, Memphis, TN, United States

**Keywords:** cerebellum, cognition, communication through coherence (CTC), cerebrocerebellar communication, cerebellothalamic tract, corticothalamic circuits

## Abstract

There is general agreement that cerebrocerebellar interactions *via* cerebellothalamocortical pathways are essential for a cerebellar cognitive and motor functions. Cerebellothalamic projections were long believed target mainly the ventral lateral (VL) and part of the ventral anterior (VA) nuclei, which project to cortical motor and premotor areas. Here we review new insights from detailed tracing studies, which show that projections from the cerebellum to the thalamus are widespread and reach almost every thalamic subnucleus, including nuclei involved in cognitive functions. These new insights into cerebellothalamic pathways beyond the motor thalamus are consistent with the increasing evidence of cerebellar cognitive function. However, the function of cerebellothalamic pathways and how they are involved in the various motor and cognitive functions of the cerebellum is still unknown. We briefly review literature on the role of the thalamus in coordinating the coherence of neuronal oscillations in the neocortex. The coherence of oscillations, which measures the stability of the phase relationship between two oscillations of the same frequency, is considered an indicator of increased functional connectivity between two structures showing coherent oscillations. Through thalamocortical interactions coherence patterns dynamically create and dissolve functional cerebral cortical networks in a task dependent manner. Finally, we review evidence for an involvement of the cerebellum in coordinating coherence of oscillations between cerebral cortical structures. We conclude that cerebellothalamic pathways provide the necessary anatomical substrate for a proposed role of the cerebellum in coordinating neuronal communication between cerebral cortical areas by coordinating the coherence of oscillations.

## Introduction

A defining characteristic of cerebral cortical function is interaction between multiple cerebral cortex areas forming a temporary task-specific functional network [e.g., (Damasio, 1989; Vaadia et al., 1995; Mesulam, 1998; Ayzenshtat et al., 2010)]. The formation and resolution of such task specific network involves precisely coordinated modulation of functional connectivity, defined as periods of increased correlation of neuronal activity (Aertsen et al., 1989; Vaadia et al., 1995). How functional connectivity is modulated at time scales compatible with normal brain function is not fully understood but evidence suggests a crucial role of the thalamus in coordinating functional connectivity between cerebral cortical areas in a task dependent manner (Ketz et al., 2015; Nakajima and Halassa, 2017; Schmitt et al., 2017). The term functional connectivity in essence describes the temporal correlation of neuronal activity between two structures measured as spike activity, local field potentials or using BOLD signals (Aertsen et al., 1989; Buckner et al., 2013). In a seminal publication, Pascal Fries proposed a mechanism for controlling functional connectivity between brain structures through the modulation of coherence of their neuronal oscillations (Fries, 2005), a mechanism he termed “communication through coherence” (CTC). Coherence of oscillations is a measure of how stable the phase relation between two oscillations of similar frequency is. Typically, coherence values change in a task dependent manner. One of the best studied examples of task related coherence increases occurs between the prefrontal cortex and dorsal hippocampus during decision making in spatial memory tasks (Benchenane et al., 2010; Gordon, 2011; Liu et al., 2022). The concept of communication through coherence has since received substantial support from experiments showing that coherence changes do indeed correlate with changes in the effectiveness of neuronal signal (i.e., spike) transmission (e.g., McAfee et al., 2018) and that changes in coherence are linked to specific behaviors, with memory and working memory related behaviors amongst the most thoroughly studied (Fell and Axmacher, 2011; Gordon, 2011; Brincat and Miller, 2015; Liu et al., 2022).

The concept of CTC thus provides an intriguing neuronal mechanism for modulating information flow and integration through the modulation of functional connectivity. Coherence and synchrony between cerebral cortical areas is known to critically depend on the thalamus and thalamocortical connectivity (Destexhe et al., 1999; Jones, 2001; Habas et al., 2009; Browning et al., 2015; Ketz et al., 2015; Mitchell, 2015; Hallock et al., 2016; Nakajima and Halassa, 2017). What is unknown, however, is how changes in coherence are controlled. Besides its massive interconnection with the cerebral cortex, the thalamus is also the key relay station for interactions between the cerebellum and the cerebral cortex (Allen and Tsukahara, 1974; Angaut et al., 1985; Habas et al., 2019). New anatomical studies have revealed that projections from the cerebellum to the thalamus are far richer and more widespread than previously believed and include numerous thalamic nuclei involved in cognitive functions (Habas et al., 2019; Fujita et al., 2020; Pisano et al., 2021). Considering the crucial role of the thalamus in modulating coherence and synchrony between cerebral cortical areas (Destexhe et al., 1999; Jones, 2001; Habas et al., 2009; Browning et al., 2015; Ketz et al., 2015; Mitchell, 2015; Hallock et al., 2016; Nakajima and Halassa, 2017), cerebellothalamic projections provide a robust interface for the cerebellum modulate thalamic activity and thus shape thalamocortical interactions. Here we review (1) evidence for the role of the thalamus in coordinating synchrony and functional connectivity between cerebral cortical areas, (2) recent literature that revealed rich projections from the cerebellum to nearly all subnuclei of the thalamus and (3) the evidence of a cerebellar involvement in coordinating coherence of oscillations in the cerebral cortex. We will focus on cerebellothalamic pathways that are likely to be involved in spatial working memory and will review a proposed new function of the cerebellum in the task-dependent coordination of functional connectivity between the medial prefrontal cortex (mPFC) and the dorsal hippocampus. The mediodorsal nucleus (MD) and nucleus reuniens (RE) of the thalamus deserve particular attention in this context due to their dense reciprocal connections to the prefrontal cortex and the reported role of the RE in coordinating coherence between the mPFC and hippocampus (Vertes et al., 2007; Browning et al., 2015; Ito et al., 2015; Ketz et al., 2015; Mitchell, 2015).

## Cerebellothalamic pathways to support sensorimotor and cognitive functions

The thalamus can be divided into two major regions–the dorsal region, containing anterior, lateral, medial, and posterior groups of nuclei, and the ventral region, made up of the thalamic reticular nucleus (TRN). The dorsal region is made of both glutaminergic projections and GABAergic interneurons, that receive input broadly from the cortex, subcortical structures, areas of the brainstem, and the cerebellum and project to localized areas of the cortex and striatum. The TRN only receives collateral projections from thalamocortical and corticothalamic neurons involved in somatosensory, sensory, and motor processes and provides exclusively GABAergic input to the dorsal thalamus (Habas et al., 2019). Traditional views associated the cerebellum solely with sensorimotor and vestibular functions and the pathways from the cerebellum to the thalamus were thought to be limited to projections from the cerebellar nuclei (CN) to the ventral or motor thalamus–specifically to the ventrolateral (VL) and parts of the ventral anterior (VA) nuclei. Recent comprehensive tracing studies have revealed far more extensive connections between the CN and the thalamus, including thalamic nuclei involved in cognitive functions (Bohne et al., 2019; Fujita et al., 2020; Pisano et al., 2021).

Bohne et al. (2019) confirmed dense projections between the fastigial, interposed, and dentate cerebellar nuclei and VL but also found new projections in the laterodorsal thalamic nucleus. Tracing experiments by Fujita et al. (2020) discovered broad connections between the fastigial nucleus of the cerebellum to several subnuclei throughout the thalamus, including the MD, VL, VM, and centrolateral (CL), and parafascicular nuclei. Pisano et al. (2021) performed a detailed study of cerebellothalamocortical pathways using trans-synaptic tracing methods. Their results also show CN projections to multiple thalamic nuclei outside of the ventral thalamus, including the MD, TRN, lateral posterior nucleus, lateral and medial geniculate nuclei, and zona incerta. Tracing experiments primarily targeted the dentate, with some expression in the interposed and fastigial nuclei. Retrograde tracing studies found axons from both the dentate and interposed nuclei in the TRN.

These findings that cerebellar projections from all three cerebellar nuclei target the thalamic nuclei involved in cognitive functions such as the MD, CL, and TRN align well with the now substantial evidence of cerebellar cognitive and affective functions (Schmahmann, 2004; Ito, 2008; Buckner, 2013; Liu et al., 2022). Detailed physiological studies of the different cerebellothalamic pathways furthermore revealed substantial pathway-specific differences in cerebellar influence on thalamic target neurons. While it was known that cerebellothalamic projections were excitatory, it turns out that the impact cerebellar projections have on postsynaptic thalamic neurons varies greatly between thalamic target nuclei (Gornati et al., 2018). Gornati et al. (2018) investigated projections from the interposed nucleus to the VL, VM, and CL thalamic nuclei and found significant differences in the sizes and density of synaptic terminals and the amplitude of postsynaptic responses. For example, glutamatergic projection terminals from the CN to the VL thalamus were significantly higher in density, displayed more complex synaptic interactions, and resulted in greater excitatory post-synaptic potentials than CN projections to the CL. Different cerebellothalamic pathways also differ in the way cerebellar-receiving thalamic neurons affect neuronal activity in their respective cerebral cortical target areas. VL thalamus has been associated with parvalbumin-positive neurons, which are found more densely in sensorimotor cortices, hippocampal, and retrohippocampal regions and are associated with spatial navigation and sensorimotor skills (Miao et al., 2017; Gornati et al., 2018; Bjerke et al., 2021). Cerebellar-receiving VL nucleus cells can also be categorized as “driver” inputs to the cortex, which further indicates a role in information processing and ongoing activity adaptation. While cerebellar projections to the VM and CL nuclei did not display significant synaptic differences from each other, the thalamocortical projections from the VM and CL nuclei are markedly different from those of the ventrolateral thalamic nuclei both in projection patterns and binding protein (Gornati et al., 2018). For example, VM and CL thalamic nuclei contain higher densities of calbindin-positive neurons and project to brain regions involved in behavior and emotion, including the infralimbic cortex, ventral tegmental area, anterior cingulate cortex, midbrain raphe nuclei, and periaqueductal gray (Van der Werf et al., 2002; Bjerke et al., 2021).

Taken together these findings show that cerebellothalamocortical pathways seem to involve most if not all of the thalamic nuclei, fitting with the rich repertoire of cerebellar motor, cognitive and affective functions. Our understanding of the physiological properties and differences between these pathways is in its infancy and an in depth investigation is essential to any attempt at understanding cerebellar contribution to brain functions.

## Sensorimotor functions

Sensory feedback continuously informs motor planning, and the cerebellar contribution mostly from the medial and interposed nuclei to this ongoing process provides a concrete example of its influence on motor cortical areas *via* the thalamus, which can be evaluated through effective execution of movements. Looking to the vibrissal system of rodents as a well-characterized model system where sensory input and motor output can be ascertained, the effect of sensory feedback on motor planning is made apparent by changes in whisking behavior as an animal encounters a tactile stimulus. Rodents tend to perform slow, low-amplitude sweeps with their whiskers in familiar environments, but then transition to rapid high-amplitude sweeps in a novel environment or when a novel stimulus is encountered (Arkley et al., 2014). For this behavioral adaptation to be effective however, there must be a mechanism for streams of sensory information to reach cortices responsible for motor planning and execution.

There are three (non-mutually exclusive) mechanisms established in functional and neuroanatomical descriptions of the rodent vibrissal system that allow sensory information to reach the motor cortex. First, a direct pathway between whisker sensory cortex (vS1) and the facial nuclei for whisker retraction allows for sensory input during whisker protraction to directly initiate retraction behavior (Matyas et al., 2010). This primes the system for more rapid protraction and active sensing when there is an object in the vibrissal field to be explored. Second, vS1 activates motor cortices for whisker retraction *via* cortico-cortical projections (Matyas et al., 2010; Mao et al., 2011), after vS1 itself is excited by tactile input. And third, neurons of cerebellar crus I and II integrate sensory and motor information streams *via* pontine and trigeminocerebellar mossy fiber inputs, and convey this combined sensorimotor information to whisker motor cortex (vM1) by way of the VL thalamic nuclei (Proville et al., 2014).

Importantly, experiments have shown that both the second and third mechanisms rely on cerebellar modulation of thalamic activity for effective somatomotor integration. In the less-obvious case of cortico-cortical communication between vS1 and vM1, synchronous rhythms between structures that promote this form of communication require an intact cerebellum (Popa et al., 2013; Lindeman et al., 2021). Using various methods to inhibit the cerebellar nuclei, it has been shown that cerebellar inactivation reduces the firing rate in motor thalamic neurons (Popa et al., 2013), decreases gamma-rhythmic coherence between vS1 and vM1 (Popa et al., 2013; Lindeman et al., 2021), and impairs the ability of animal to adapt whisking strategies appropriately in a changing sensory context (Proville et al., 2014).

Execution of head, limb, eye, or truncal movements may rely on different or additional pathways for sensory feedback in motor planning, but the available evidence suggests that cerebellothalamocortical pathways are crucial for the planning and execution of these movements as well. Each of these somatic regions exhibit robust representation within the cerebellum (Grodd et al., 2001; Manni and Petrosini, 2004; Grimaldi and Manto, 2012), with evidence of integrated sensory and motor representations (Wiestler et al., 2011). Somatic areas exhibit robust correlation with corresponding regions of the motor cortex (Buckner et al., 2011; Saadon-Grosman et al., 2022), which are connected *via* thalamic nuclei. Therefore, the cerebellar contribution to motor planning in other somatic domains is likely to be similar in principle to the mechanisms outlined here, albeit more complex with the execution of more complex movements in a more spatially complex environment.

## Cognitive functions

In the following two sections we will discuss the roles of the MD thalamic nucleus and the nucleus reuniens (RE) in shaping cerebral cortical activity and cognitive function. The MD nucleus prominently projects to the mPFC, an area of cerebral cortex widely associated with cognitive function (Guldin et al., 1981) and these projections allow patterns of prefrontal activity to persist when task-relevant information needs to be held in mind. The RE has been shown to play a key role in coordinating the coherence of neuronal oscillations, and hence the functional connectivity, between the medial prefrontal cortex and the hippocampus (Hallock et al., 2016) which is critical for spatial working memory (SWM) and navigation.

### Mediodorsal nucleus

As a general rule, thalamic activity directs the flow of information to and throughout the neocortex. For lower sensory cortical areas, the role of the thalamus is manifest as a sort of sensory relay station, where thalamic impulses modulate excitability at appropriate times and convey specific sensory information to cortical neurons. For higher-order areas like the PFC, the thalamus conveys no specific information, but instead seems to modulate the tone of cortical neurons in a manner that is topographically selective and precisely timed for the gating and maintenance of task-relevant information (Mitchell, 2015; Schmitt et al., 2017; Honjoh et al., 2018). The mediodorsal (MD) thalamic nucleus is interconnected with the prefrontal cortex in mammals (Guldin et al., 1981; Ray and Price, 1993; Kuramoto et al., 2017), and the timing of MD activity affects the flow of information on two different timescales. First, on the order of hundreds of milliseconds, increased MD activity signals that sensory information is being presented which is relevant to gain a future reward. On the timescale of milliseconds, sustained MD activity drives fast interneuron rhythms while disinhibiting principal neurons (Anastasiades et al., 2020), promoting communication between principal neurons that signal coherently with the inhibitory rhythm. The result, as demonstrated by Schmitt et al. (2017) is thalamic activity that promotes precisely timed communication between cortical neurons that are tuned to common information, which is thought to help sustain the neuronal representation of that information during a delay period.

How the MD is activated on the broader timescale in an appropriate manner for a given task has not yet been fully explored. Some of this thalamic recruitment is thought to occur as a top-down phenomenon, initiated by the PFC itself when conscious effort is made to maintain information in mind for decision-making. The cerebellum is well positioned to assist in the task-relevant modulation of MD as well, and likely plays a role that is mechanistically similar to how it promotes functional connectivity within the sensorimotor system. In rodents, the fastigial nuclei project to the lateral MD thalamic nuclei (Fujita et al., 2020), which in turn project to the prelimbic prefrontal cortex to modulate inter-neuronal communication (Divac et al., 1993; Kuramoto et al., 2017). Functional circuit mapping techniques in rodents have shown that projections to the cerebellum (*via* the pons) from the prelimbic PFC predominantly terminate within the lateral vermis (Watson et al., 2009), which in turn project to the fastigial nuclei to close the circuit (Fujita et al., 2020). Given the known anatomy, the cerebellum could have a supportive role in recruiting the MD nuclei in response to PFC activity. Additionally, the vermal cortex that provides input to the fastigial nuclei may serve as a substrate for potential predictive activation of MD in the appropriate sensory context, but further information is needed as to what sources of input converge in the vermis.

In humans, the prefrontal cortices and neocerebellum are selectively expanded in comparison to rodents (Balsters et al., 2010), and primates show more numerous and extensive pathways connecting the cerebellum, thalamic nuclei, and prefrontal cortex. In human imaging studies, MD shows a broader functional relationship with the cerebellar hemispheres, which is notably diminished in patients with schizophrenia (Anticevic et al., 2014).

### Nucleus Reuniens

We focus on the mPFC and hippocampus because several independent studies have shown that SWM requires the coordinated activity of the mPFC and dorsal hippocampus (Churchwell and Kesner, 2011; Gordon, 2011). Simultaneous electrophysiological recordings in the mPFC and hippocampus during performance of SWM tasks have shown that the decision process is associated with an increase in the coherence of theta oscillations between the mPFC and dorsal hippocampus (Jones and Wilson, 2005; Hyman et al., 2010; Benchenane et al., 2011; Gordon, 2011; Liu et al., 2022). A comparison of correct and incorrect decisions revealed that mPFC-hippocampal theta coherence reached higher values during correct compared to incorrect decisions, supporting a functional role of coherence in this task (Jones and Wilson, 2005; Hyman et al., 2010; Liu et al., 2022). Coherence of neuronal oscillations does not impact brain function unless it affects changes in spike activity within the communicating regions. It is important to note that in the context of SWM two studies measured both spike activity and local field potential (LFP) coherence and showed that an increase in coherence was accompanied by an increase in entrainment of mPFC spike activity to the phase of the coherent mPFC-hippocampal theta oscillations (Jones and Wilson, 2005; Hyman et al., 2010). For additional examples of experimental support an influence of coherence on spike activity see also (Jones and Wilson, 2005; Siegel et al., 2008; Bosman et al., 2012; Brunet et al., 2014; Sigurdsson and Duvarci, 2016; Bonnefond et al., 2017; Palmigiano et al., 2017; McAfee et al., 2018). Thus, changes in coherence between the mPFC and hippocampus are strongly implicated in SWM. The thalamic nuclei involved in controlling mPFC-hippocampal coherence could thus serve as the interface for cerebellar contributions to SWM decision making which involves cerebellar lobulus simplex as recently reported in mice performing a SWM task (Liu et al., 2022).

When considering the influence of the cerebellar cortex on mPFC-hippocampal coherence during SWM decision making, the RE is a possible key thalamic nucleus involved in the modulation of that coherence. Neurons in the RE receive excitatory inputs from the prelimbic and infralimbic areas of mPFC and in turn send dense excitatory projections to dorsal CA1 region of the hippocampus (Vertes et al., 2007). While direct ventral hippocampal projections to mPFC had already been established (Ferino et al., 1987; Carr and Sesack, 1996) this tracing study showed that hippocampal-prefrontal connectivity was in fact reciprocal *via* the RE. Additional tracing studies have also identified populations of RE cells that send collaterals to both mPFC and hippocampus (Hoover and Vertes, 2007; Varela et al., 2014), establishing bidirectional connectivity between mPFC and RE. The functional implications of this pathway have since been further explored in the context of working memory (Hallock et al., 2013; Ito et al., 2015).

Dolleman-van et al. (2019) and Griffin (2021) wrote comprehensive reviews about the role of RE in coordinating hippocampal-prefrontal interactions during working memory. One report central to both these reviews was a study from Ito et al. (2015) which showed that neurons in the mPFC, RE, and CA1 in rats exhibited trajectory-dependent firing in a continuous alternation task using a modified T-maze. Trajectory-dependent firing is a key component to spatial navigation in that it contains predictive information about future positions as well as instantaneous position, which is crucial to establishing a “goal path.” Permanent inactivation of RE *via* lesions significantly impaired trajectory-dependent firing in CA1. Transient optogenetic inactivation of RE neurons also led to a significant decrease of trajectory-dependent firing in CA1. This study shows that projections from mPFC to CA1 *via* RE are crucial for trajectory-dependent firing in CA1 and provides additional evidence for the role of thalamic nuclei in coordinating long-range communication between cortical regions (Ito et al., 2015).

In addition to RE’s role in facilitating trajectory-dependent firing, new work has shown that RE contributes to the coordination and stabilization of neuronal assemblies within mPFC and hippocampus (Angulo-Garcia et al., 2020). In experiments using anesthetized rats and *in vivo* electrophysiology, it was shown that assemblies of RE neurons activate sequentially during “up states” of slow LFP oscillations, which preceded activation of mPFC assemblies. “Up states” are defined as the periods from the peak to the trough of the filtered slow oscillation LFP signal. Chemogenetic inactivation of RE disrupted mPFC assembly onset during up states as well as hippocampal assemblies present during sharp wave ripples. The authors suggest that RE may be necessary to stabilize mPFC and hippocampal cell assemblies. This report provides further evidence that RE is a functional hub for prefrontal-hippocampal interactions (Angulo-Garcia et al., 2020).

We currently know little about cerebellar projections to RE. The most detailed recent tracing studies suggest that projections exist but might be sparse (Fujita et al., 2020; Pisano et al., 2021). More focused studies are required to determine the extent and physiological effectiveness of cerebellar influence on the RE.

## Summary

Understanding the broad involvement of the cerebellum in motor, affective and cognitive brain function it is essential to gain a detailed understanding of the pathways that connect the cerebellum with the cerebral cortex *via* the thalamus. We have reviewed rich new evidence showing that cerebellar projections from all three cerebellar nuclei seem to reach most, if not all nuclei of the thalamus and that each of these pathways may have unique physiological properties in terms of cerebellar influence on thalamic neurons and in terms of the influence of cerebellar receiving thalamic neurons on the cerebral cortex. Clearly, in order to understand the role of the cerebellum in its various functions that require cerebrocerebellar interactions, the cerebellothalamocortical pathways must be a major focus of future investigations.

## Author contributions

DH developed the concept of the review. DH, MF, BC, SM, and YL jointly wrote and edited the manuscript. All authors contributed to the article and approved the submitted version.
